# Coupling the effects of extreme temperature and air pollution on non-accidental mortality in Rencheng, China

**DOI:** 10.3389/fpubh.2023.1241385

**Published:** 2023-08-03

**Authors:** Daozheng Yu, Soo-Beom Lee, Si Chen, Seong Wook Kim, Shuaishuai Xi

**Affiliations:** ^1^School of Resources and Environmental Science, Hubei University, Wuhan, China; ^2^Department of Transportation Engineering, University of Seoul, Seoul, Republic of Korea; ^3^Department of Mathematical Data Sciences, Hanyang University, Ansan, Republic of Korea; ^4^Center for Disease Control and Prevention, Rengcheng, Jining, China

**Keywords:** extreme temperature, air pollutants, non-accidental deaths, relative risks, distributed lag nonlinear model

## Abstract

**Background:**

Extreme temperatures and air pollution have raised widespread concerns about their impact on population health.

**Aim:**

To explore the quantitative exposure risks of high/low temperatures and types of air pollutants on the health of various populations in urban areas in China, this study assessed the effects of temperature and air pollutants on daily non-accidental deaths in Rencheng District, Jining City, China from 2019 to 2021.

**Methods:**

A combination of Poisson regression models and distributed lag non-linear models was used to examine the relationships between temperature, air pollutants, and daily non-accidental deaths. We found that temperature and air pollutants had a significant non-linear effect on non-accidental mortality. Both high and low temperatures had a noticeable impact on non-accidental deaths, with heat effects occurring immediately and lasting 2–3 days, while cold effects lasted for 6–12 days. The relative risks of non-accidental deaths from PM_2.5_, NO_2_, and SO_2_ were highest in winter and lowest in autumn. The relative risk of non-accidental deaths from O_3_ was highest in spring, with no significant variations in other seasons. Older adults (≥75) and outdoor workers were at the greatest risk from temperature and air pollutant exposure.

**Conclusions/interpretation:**

Exposure to extreme temperatures and air pollutants in the Rencheng District was associated with an increased mortality rate. Under the influence of climate change, it is necessary for policymakers to take measures to reduce the risk of non-accidental deaths among residents.

## 1. Introduction

Climate change and air pollution are serious threats to human health (1), economies, and environments across the world. Over the past 50 years, human activities especially in areas, such as industry, transportation, power generation, and fossil fuel combustion have led to the emission of large amounts of greenhouse gases and air pollutants, trapping heat and pollutants in the atmosphere to exacerbate atmospheric warming and decrease air quality. A comprehensive report compiled in the sixth assessment report of the Intergovernmental Panel on Climate Change completed by three working groups pointed out that the current global average surface temperature is about 1.1 degrees Celsius higher than pre-industrial levels, and global surface temperatures will continue to increase until at least the middle of this century (2). If greenhouse gas emissions are not reduced in the future, global temperatures will likely exceed 1.5 or 2°C by the end of the 21st century (3). Along the same timescale, the World Health Organization reported that 99% of the world’s population lives in places that do not meet their air quality guidelines, resulting in about 7 million deaths worldwide each year from air pollution and millions of years of healthy life lost (4, 5). Up to one-third of deaths caused by stroke, lung cancer, and heart disease can be attributed to air pollution (6). With an increase in global temperatures and the universality of air pollution, the intensity and frequency of extreme temperatures and high concentration pollutant events are increasing rapidly.

Most of the world is suffering from extreme temperatures and high concentrations of air pollutants, which have had a significant impact on human health. Some epidemiological studies have illustrated that exposure to extreme temperatures and high concentrations of air pollutants can result in serious cardiovascular, cerebrovascular, and respiratory diseases and even mortality (7). More than 5 million people worldwide died each year from heat or cold between 2000 and 2019 (8). According to a report released by Reuters in July 2021, there were 486 premature deaths within a 5-day period caused by high temperatures in British Columbia, Canada (9). In Spain from July 10 to July 17, 2022, and Portugal from July 7 to July 18 in 2022, there were 678 and 1,063 premature deaths due to heat, respectively (10). The CCTV News Agency reported on February 6, 2012 that 112 people died from cold-related deaths in Ukraine (11, 12). Meanwhile, air pollution has become the fifth leading cause of death worldwide. According to data from the World Meteorological Organization in 2021, the number of additional deaths caused by outdoor air pollution increased from 2.3 million in 1990 to 4.5 million in 2019 (6). A study by Lancet showed that, in 2019, new asthma cases in 1.85 million children worldwide were associated with NO_2_, which accounted for 8.5% of all new asthma cases reported in children that year (13). In 1973 to 2018, Meng et al. (14) found that, for every 10 μg/m^3^ increase in SO_2_ concentration, the non-accidental mortality rate increased by 0.3% based on a study conducted in 22 European countries. Exposure to extreme temperatures and high concentrations of air pollutants may not only lead to health risks of premature death, but also impose a burden on the economy. A study on global extreme high temperatures caused by climate change showed that, from 1992 to 2013, extreme high temperatures caused global economic losses of at least 16 trillion dollars (15). The Swiss Reinsurance Institute released a report that, by 2050, the global economy may lose 10% in GDP due to climate change (16). As estimated from a study in Lancet, pollution-related diseases resulted in health-care costs that were responsible for 1.7% of annual health spending in high-income countries and for up to 7% of health spending in rapidly developing and heavily polluted middle-income countries (17).

While the inter-relationship between air pollution and temperature extremes is widely known and well documented, their combined effect on the target organs of human beings could exacerbate the risk of adverse conditions and lead to premature death, promoting the need to investigate the impact of interactions between ambient air pollution and temperature extremes on health and mortality (18, 19). Qian et al. (20) found an interaction between high temperatures and PM_10_ in Wuhan City in China, which synergistically promoted an increase in non-accidental population mortality. Stafoggia et al. (21) showed a strong association of temperature and air pollutants on mortality in a study of several Italian cities. In 2010, the interaction of heat and air pollution due to catastrophic heat waves and wildfires in Moscow, Russia caused more than 2,000 additional deaths compared with deaths in the same period from 2006 to 2009 (22). In addition, seasonality is a potentially important confounding factor in the study of the impacts of air pollution and extreme temperature on mortality, which may significantly result in variations in mortality rates. However, due to differences in peak concentrations and compositions of environmental pollutants, temperature, humidity, and economic development levels, the results of seasonal differences in the correlation between air pollution and mortality are quite different. Peng et al. (23) found that PM_10_ values in the Northeast exhibited strong seasonal variation (peaking in summer), while seasonal variations in PM_10_ in the South were minimal. Meanwhile, the correlation between air pollution and mortality in the summer was significantly higher than in the winter in Western Europe and Seoul City, Korea (24, 25). In contrast, Zhu et al. (26) believed that ambient air pollution had a greater impact on acute mortality during the cold season in Wuhan City, China. In addition to a study on the relationships between temperature, pollution, and mortality, the time lag effect should be considered for greater accuracy. The European Air Pollution Health Program (APHEA), which began in 1993, has quantitatively estimated the short-term effects of air pollution on human health, showing a correlation between pollutants and non-accidental deaths with an observed existence of short-term lagged effects (27–29). These lagged effects were also found in a study based on 20 cities in the United States, which showed a high associated of PM_10_ with heart disease deaths after a 2-day lag (30). Qian et al. studied seven cities in China (Beijing, Tianjin, Xi’an, Harbin, Shanghai, Guangzhou, and Wuhan) and found that the lower was the temperature, the longer was the duration of the effect, generally lasting for 2 weeks; the maximum effect value appeared within 3–5 days (20). The effect of high temperature was short-lived, with the maximum effect estimated on the day of exposure or with a lag of 1–2 days (31). Due to higher vulnerability and limited capacity to adapt to extreme temperatures and air pollutants, developing countries are more likely to sustain health threats associated with extreme temperatures and air pollutants than are developed countries. As a developing country, China has been the subject of few comprehensive research studies exist on the serious impact of extreme temperatures and air pollution on population health, which is an urgent need for analysis especially in non-metropolitan cities.

In addition, China has been the focus of few comprehensive research studies on the serious impact of extreme temperatures and air pollution on population health and seasonal differences in industrial cities (especially non-metropolitan cities); therefore, it is appropriate to evaluate the non-accidental deaths caused by extreme temperatures and air pollution from 2019 to 2021 in Jining, the most populous of China’s top 10 coal cities.

The health effects of exposure factors such as air pollution and meteorology exhibit persistence and time lag. Various methods exist to evaluate the lag effects of exposure factors such as the moving average method, generalized linear model, and generalized additive model (32). Most methods only consider the effect over a specific period and the corresponding exposure level of several consecutive days, neglecting the characteristics of a lag distribution and resulting in high collinearity and non-negligible deviation. Application of a distributed lag linear model (DLM) has led to great progress in the study of this problem, but the model is limited to the linear exposure-response relationship. The relationship between exposure and response is nonlinear for most cases such as the U-shaped, J-shaped, or V-shaped distribution curves for temperature. On this basis, Armstrong introduced a distributed lag nonlinear model (DLNM) for study of health effects of temperatures in 2006 to identify the nonlinear influence of time lag (33, 34). The model is not limited to the study of the effects of air pollution or temperatures on human health, but can also be extended to any time series study exploring relationships between predictive variables and outcomes as well as lagging effects, covering many fields such as climate and environmental studies, public health, and economics. In this study, we employed a DLNM method to investigate the relationships between temperature, air pollutants, and non-accidental deaths considering nonlinear time lag effects. An industrial city in Shandong Province, Jining City, which is a coal city with large population, was selected as for the analysis. The purpose of this paper is to (1) explore the interactions and seasonal differences between extreme temperatures and air pollutants; (2) investigate the roles of extreme temperatures and air pollutants in non-accidental mortality and identify the most vulnerable groups; (3) propose management strategies to assist policy makers in establishing early warning systems to reduce extreme temperature and air pollution exposure, especially of vulnerable populations, and improve the quality and level of public health management for the study area.

## 2. Materials

### 2.1. Study area and data sources

**Figure 1:**
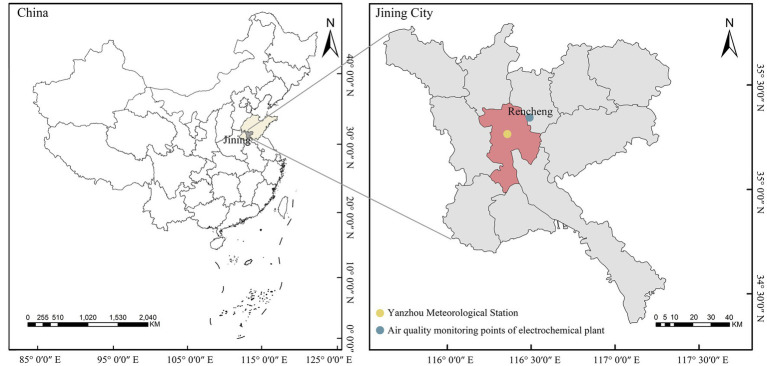
The geographical location of the Rencheng District.

The Rencheng District of Jining City is located in the southwest of Shandong Province in China (35°08′N to 35°32′N, 116°26′E to 116°44′E), with a total area of 651 km^2^ and about 1.0756 million residents (shown in Figure 1). Located in the East Asian monsoon climate zone, Rencheng District is hot and rainy in the summer and cold and dry in the winter. From 1970 to 2014, there were 383 days where the daily maximum temperature in Jining exceeded 35°C and 243 days with a minimum temperature below −10°C. As the largest coal industrial base in Shandong Province and a top 10 coal city in China, Rencheng has poor air quality. From 2019 to 2021, there were 373 days of light pollution, 103 days of moderate pollution, and 24 days of heavy pollution in Rencheng District. A study of the severe effects of extreme temperatures and air pollution on population health in Rencheng District is expected to provide data to local policy makers to mitigate the health losses caused by extreme temperatures and air pollution.

In the current study, daily mortality counts from the Rencheng district of Jining between January 2019 and December 2021 were collected from the Jining Center for Disease Control and Prevention. The non–accidental death data comprised 21,647 cases in the Rencheng District during the study period and were classified according to gender (male, female), age (0–59, 60–74, ≥75), and work environment (indoor worker, outdoor worker). In the working environment, according to the occupational classification of the People’s Republic of China, we adjusted the occupational system of the People’s Republic of China into eight categories, and divided them into indoor workers and outdoor workers according to the characteristics of work. Among them, the indoor workers included: (1) the heads of state organs, party organizations, enterprises, and public institutions, (2) professional and technical personnel, (3) clerks and related personnel, (4) social production service and life service personnel; while the outdoor workers included: (1) agricultural, forestry, animal husbandry, fishery production, and auxiliary personnel, (2) production, transportation operators, and related personnel, (3) military personnel, and (4) other employees who are inconvenient to classify.

Daily air pollutant concentrations, [i.e., sulfur dioxide (SO_2_), nitrogen dioxide (NO_2_), fine particulate matter (PM_2.5_), and ozone (O_3_; μg/m^3^)] from the air quality monitoring station of Jining were collected from the national urban air quality real-time publishing platform.^1^ Daily temperature datasets, including daily maximum temperature, daily minimum temperature, and daily average temperature (°C), were collected from the National Meteorological Science Data Center.^2^ The locations of an air quality monitoring station (1655A) and a meteorological station (54916) in the Jining District are shown in Figure 1.

### 2.2. Method

The effects of temperature and air pollutants on mortality depend not only on immediate exposure on a given day, but also on exposure over the several preceding days (lag effect). Thus, considering the simultaneously nonlinear and lag effects of temperature and air pollutants on non-accidental mortality, we employed a distributional lag nonlinear model (DLNM) to evaluate the exposure response relationship. In order to capture the lagged and non-linear associations, a corresponding cross-basis for the observed time series of each influencing factor was established to specify the exposure-lag-response dependency simultaneously in the current and lagged dimensions. The day of the week (DOW) was set as a dummy variable to control the influence of pollutant emission cycle characteristics. The DLNM is as follows:


(1) $$\log\left[E\left(Y_{t}\right)\right]=\alpha +\overset{p}{\underset{p=1}{\sum }}cb.S_{p}\left(X_{p,ti};\theta_{p,i}\right)+S\left(time_{i},\delta_{i}\right)+DOWt$$

where *t* is the observation day, *Y_t_* denotes the observed daily mortality on calendar day *t*, and *α* is the intercept. The variable *cb.s_p_*(*X_p,ti_*; *θ_p,i_*) denotes the transformed cross-basis of the *p*-th influencing factor *X_p,ti_* [i.e., maximum temperature (T_max_; °C); minimum temperature (T_min_; °C); average temperature (T_ave_; °C); levels of sulfur dioxide (SO_2_), nitrogen dioxide (NO_2_), fine particulates (PM_2.5_), and ozone (O_3_) in this study], specified through a cross-basis function with parameters *θ_p,i_* [i.e., degree of freedom (dof), 7 in this study] for both the predictor and lag dimension. The variable *S*(time_i_; *δ_i_*) denotes the transformed basis of time specified by a natural cubic B-spline function with parameters *δ_i_* (which were set to 7) used to remove unmeasured long-term and seasonal trends in the time series. *DOW* is the effect of the day of the week as a categorical variable. The cross-basis of DLNM was used for exploring and modeling the non-linear and distributed lag structures of temperature and atmospheric pollutant effects over a lag of 0–14 days; natural cubic splines with dof = 3 were used to account for spaces of *X_p,ti_*; and spline knots were inserted at quantiles (25, 50, and 75%) in the log scale of *X_p,ti_* to estimate the cumulative effects of various values of *X* (32, 35).

## 3. Results

### 3.1. Descriptive statistics of temperature, air pollution, and non-accidental deaths

#### 3.1.1. General statistics

Table 1 summarizes the basic statistics for non-accidental mortality, temperature, and air pollutant concentrations in the Rencheng District from 2019 to 2021. During the study period, the daily mean concentrations were 51.8 μg/m^3^ for PM_2.5_, 32.67 μg/m^3^ for NO_2_, 12.93 μg/m^3^ for SO_2_, and 71.34 μg/m^3^ for O_3_. According to the current air quality standards in the residential area of China, the annual standard values are 35 μg/m^3^ for PM_2.5_, 40 μg/m^3^ for NO_2_, 60 μg/m^3^ for SO_2_, and 160 μg/m^3^ for O_3_. The PM_2.5_ concentration exceeded the annual mean level of the national grade II, while the SO_2_, NO_2_, and O_3_ concentrations did not exceed this level. The annual average values of the daily minimum, average, and maximum temperatures were 9.77, 15.02, and 20.79, respectively. During the study period, a total of 21,647 non-accidental deaths occurred in the Rencheng District. On average, there were 20 cases per day, including 11 deaths in men and nine deaths in women; 11 deaths in indoor workers and nine deaths in outdoor workers; and three deaths in people aged 0–59 years, five deaths in people aged 60–74 years, and 12 deaths in people aged >75 years.

**Table 1:** Distributions of non-accidental mortality, temperature, and air pollutants from 2019 to 2021 in Rencheng.

Variable	X— ± S	Min	Percentile			Max
			P25	P50	P75	
Non-accidental death						
Total death count	19.75 ± 5.72	6	16	19	23	52
Male	10.967 ± 3.76	1	8	11	13	28
Female	8.78 ± 3.5	1	6	8	11	29
Indoor worker	10.5 ± 2.91	5	9	10	12	26
Outdoor worker	9.376 ± 4.49	1	6	9	12	35
0–59 years	2.96 ± 1.607	1	2	3	4	10
60–74 years	5.496 ± 2.46	1	4	5	7	14
>75 years	11.5456 ± 4.31	2	8	11	14	31
Temperature						
T_min_ (°C)	9.768 ± 10.164	−16.2	0.9	10	19.2	28.5
T_ave_ (°C)	15.02 ± 9.951	−11.4	6.5	15.2	24.3	32.7
T_max_ (°C)	20.788 ± 9.92	−7.2	12.9	21.2	30	38.5
Air pollution concentration						
PM_2.5_ (μg/m^3^)	51.8 ± 34.79	4	28	42	65	241
NO_2_ (μg/m^3^)	32.67 ± 17.05	3	19	29	44	90
SO_2_ (μg/m^3^)	12.935 ± 6.45	3	9	12	16	75
O_3_ (μg/m^3^)	71.34 ± 36.695	4	41	68	96	191

#### 3.1.2. Seasonal characteristics

**Figure 2:**
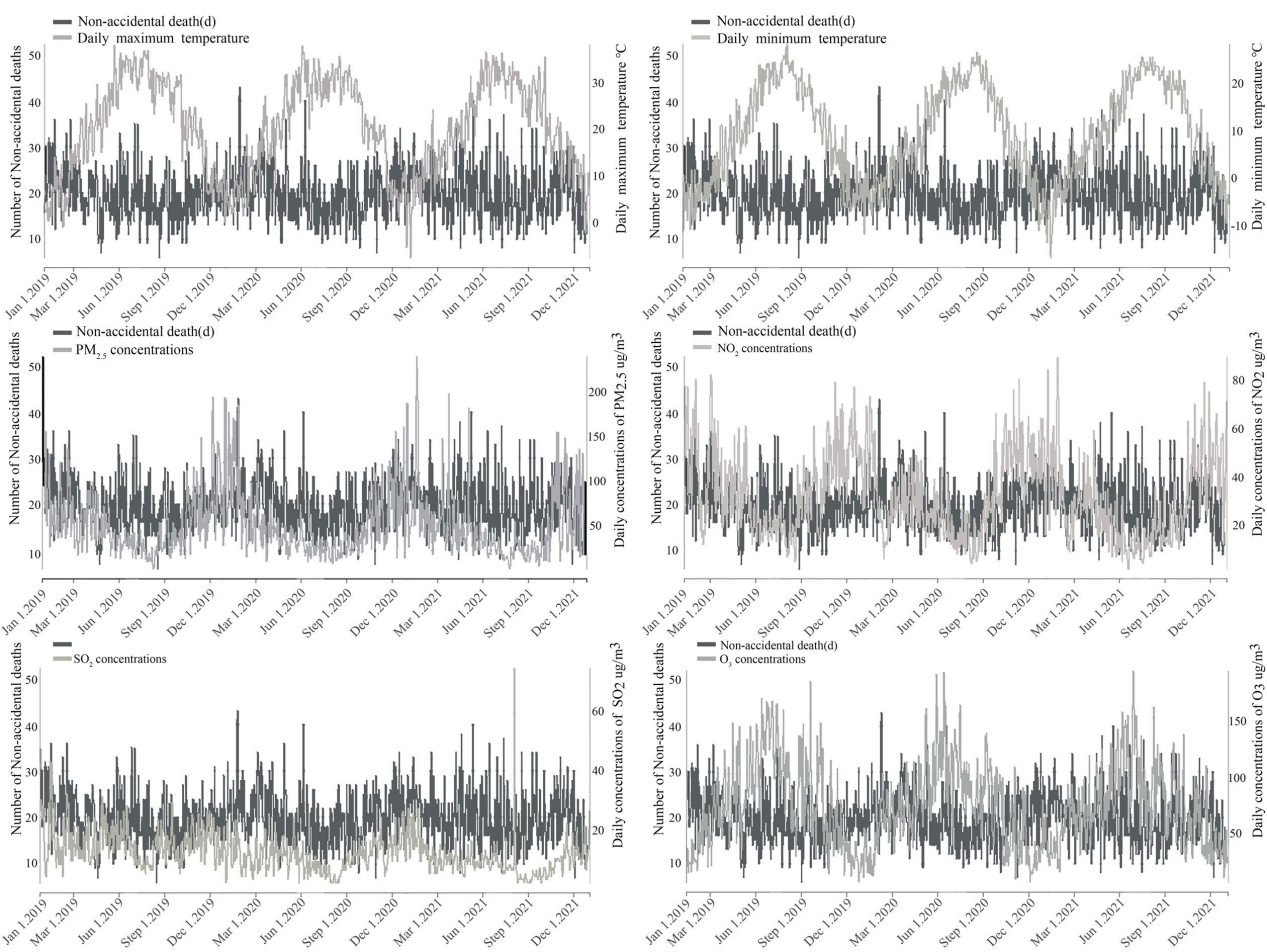
Time-series of non-accidental mortality, temperature, and air pollution concentrations in Rencheng.

Table 2 and Figure 2 show the seasonal variations of temperature (daily minimum temperature, daily average temperature, and daily maximum temperature), air pollutant (PM_2.5_, SO_2_, NO_2_, and O_3_) concentrations, and non-accidental deaths in the Rencheng District from 2019 to 2021. Non-accidental deaths showed a seasonal pattern, with higher counts in winter and lower counts in summer. In spring, summer, autumn, and winter, the average number of daily non-accidental deaths was 20.04, 18.37, 19.2, and 21.35, respectively. Temperature and pollutant concentration showed relatively stable annual trends. Temperature and O_3_ showed a seasonal trend of high values in the summer and low values in the winter. The concentrations of PM_2.5_, SO_2_, and NO_2_ were highest in the winter and lowest in the summer. Seasonal variation in PM_2.5_ concentration was most obvious and was three times higher in the winter (81.32 μg/m^3^) than in the summer (26.8 μg/m^3^). Seasonal variation in SO_2_ concentration was the weakest and was 1.6 times higher in the winter (16 μg/m^3^) than in the summer (10.1 μg/m^3^).

**Table 2:** Concentration of air pollutants and non-accidental deaths in different seasons from 2019 to 2021 in Rencheng.

Season	Days (d)	X— ± S					T_ave_ (°C)
		Non-accidental deaths	PM_2.5_ (μg/m^3^)	NO_2_ (μg/m^3^)	SO_2_ (μg/m^3^)	O_3_ (μg/m^3^)	
Spring	276	20.04 ± 5.58	49 ± 23.5	30 ± 11.1	13.17 ± 5.14	83.5 ± 27	15.5 ± 5.5
Summer	276	18.37 ± 5.69	26.8 ± 10.4	17.5 ± 8	10.1 ± 6.42	101 ± 32.9	26.72 ± 2.42
Autumn	273	19.2 ± 5.05	50.55 ± 31.2	40.3 ± 15.63	12.47 ± 5.3	59.12 ± 32	15.47 ± 6.65
Winter	271	21.35 ± 6.08	81.32 ± 41.5	42.96 ± 18.3	16 ± 7.24	40.94 ± 28.9	2.16 ± 3.78

### 3.2. Correlation analysis of temperature, air pollutant concentration, and non-accidental deaths

The results of Spearman’s correlation analysis are shown in Table 3, where non-accidental deaths were positively correlated with PM_2.5_, SO_2_, and NO_2_. In addition, there was a significant positive correlation between PM_2.5_, SO_2_, and NO_2_. Conversely, non-accidental deaths and PM_2.5_, SO_2_, and NO_2_ were negatively correlated with O_3_ and temperature with statistical significance (*p* < 0.01). The strongest positive correlation was found between O_3_ and daily maximum temperature (*r* = 0.764), and the strongest negative correlation was found between NO_2_ and daily minimum temperature (*r* = −0.647). The strong association between air pollutants and meteorological elements suggested an inherent link between the two, and that the effects on human health were not univocal, suggesting that the interaction of temperature and air pollutants has a non-negligible health effect on the population.

**Table 3:** Spearman’s correlation coefficients between daily temperature, air pollutants, and non-accidental deaths in Rencheng.

	Non-accidental deaths	PM_2.5_ (μg/m^3^)	SO_2_ (μg/m^3^)	NO_2_ (μg/m^3^)	O_3_ (μg/m^3^)
PM_2.5_ (μg/m^3^)	0.219^*^				
SO_2_ (μg/m^3^)	0.148^*^	0.494^*^			
NO_2_ (μg/m^3^)	0.175^*^	0.631^*^	0.565^*^		
O_3_ (μg/m^3^)	−0.107^*^	−0.345^*^	−0.126^*^	−0.609^*^	
T_min_ (°C)	−0.212^*^	−0.602^*^	−0.400^*^	−0.647^*^	0.648^*^
T_ave_ (°C)	−0.205^*^	−0.593^*^	−0.327^*^	−0.625^*^	0.730^*^
T_max_ (°C)	−0.193^*^	−0.550^*^	−0.241^*^	−0.569^*^	0.764^*^

* *p* < 0.01.

### 3.3. The exposure-response association between extreme temperature and non-accidental mortality

**Figure 3:**
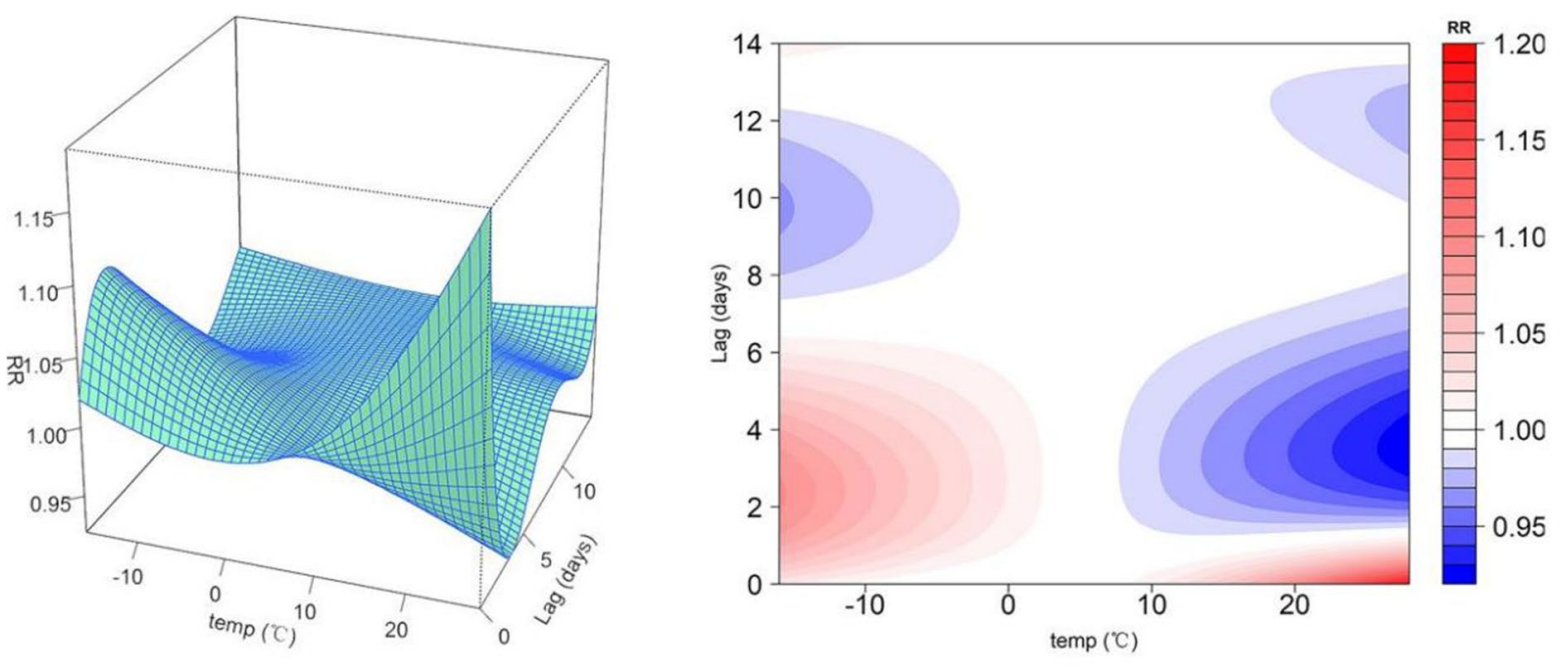
**(A)** Relative risk of mortality by daily minimum temperature over 14 lag days. **(B)** Relative risk of mortality by daily maximum temperature over 14 lag days.

Figure 3 shows two-dimensional and three-dimensional plots of the relationship between daily minimum/maximum temperature and relative risk (RR) of non-accidental mortality for the 14 lag days. From Both extreme high and extreme low temperatures led to an increased risk of non-accidental mortality; the effect on mortality tended to last longer on cold days, and the RR maximum tended to be higher on hot days. The cold effect was primarily long-term on non-accidental mortality, which tended to be strongest on lag day 2 with an RR of 1.09 and could last for about 6–12 days. However, the heat effects on non-accidental mortality were mainly short-term, with the strongest effect on lag day 0 (RR = 1.08) and lasting for about 2–3 days.

Meanwhile, the low temperature ranged from P1 to P25 (−9.4–6.5°C) and high temperature ranged from P75 to P99 (24.4–32.3°C) of the daily average temperature in Rencheng District, which were used as the temperature thresholds for the low temperature effect and the high temperature effect, respectively. The percentage of non–accidental mortality increases caused by each 1°C decrease/increase in lag 0–14 days was calculated. The results showed that when the average daily temperature varied between P1 and P25 (−9.4–6.5°C), and the non-accidental mortality rate increased by 0.56% (95% CI: −1–2%) for every 1°C decrease in temperature. Meanwhile, when the average daily temperature varied between P75 and P99 (24.4–32.3°C), the non-accidental mortality rate increased by 0.58% (95% CI: −2–3%) for every 1°C increase in temperature.

### 3.4. The exposure-response association between air pollutants and non-accidental mortality

**Figure 4:**
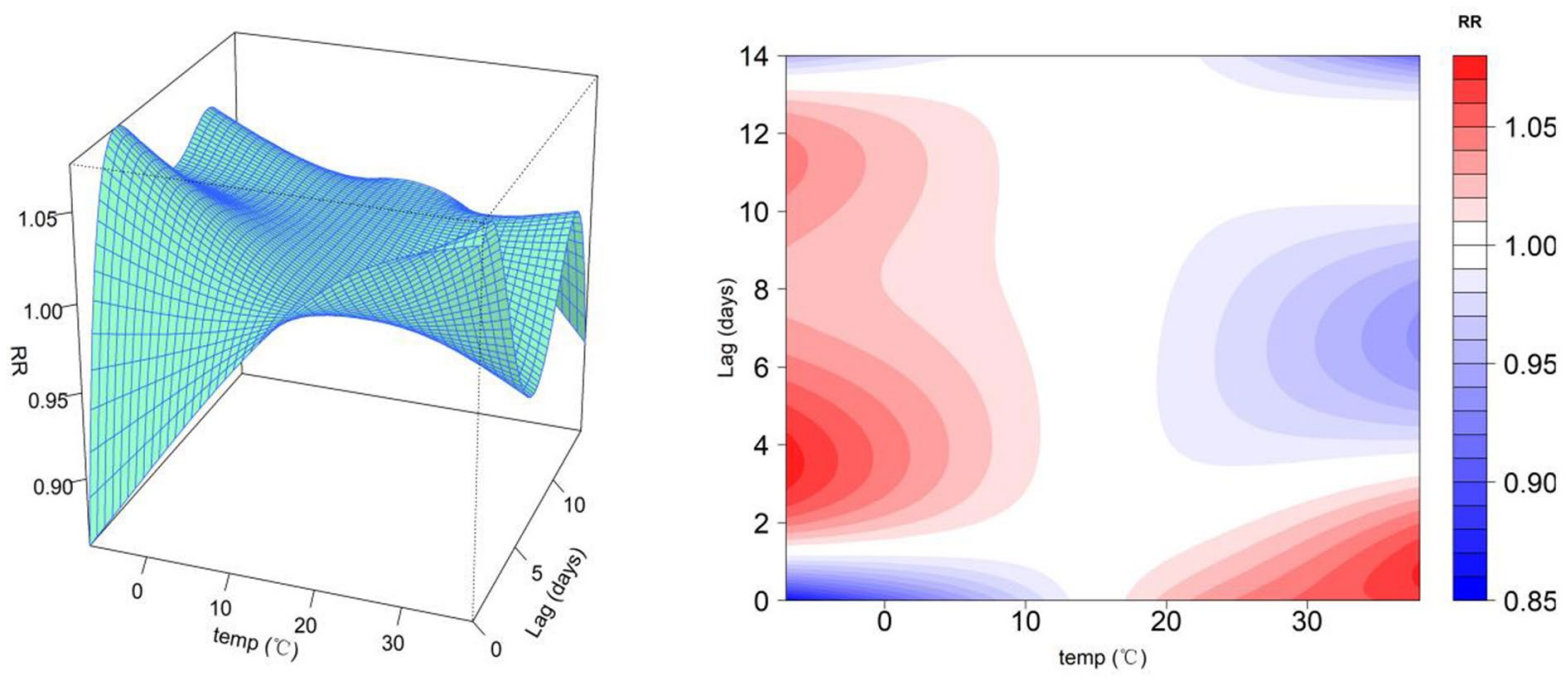
Cumulative 14-day relative lag risk of non-accidental mortality for every 10 μg/m^3^ increase in air pollution concentration.

In order to investigate air pollutants and their corresponding seasonal influence on non-accidental mortality, the cumulative RRs increased by year, and seasonal impacts of PM_2.5_, NO_2_, SO_2_, and O_3_ concentrations across 14 lag days were calculated and plotted in Figure 4. Over 1 year, the cumulative RR of non-accidental death was highest for NO_2_ exposure and tended to be less than 1 for O_3_ concentration. The cumulative RR of non-accidental death increased by 0.05 (95% CI: 0.02–0.07), 0.08 (95% CI: 0.02–0.14), and 0.04 (95% CI: −0.02–0.09) for every 10 μg/m^3^ increase in PM_2.5_, NO_2_, and SO_2_ concentration, respectively.

From the seasonal analysis, the effects of three pollutants (PM_2.5_, SO_2_, and NO_2_) on non-accidental death were consistent with the concentration variation over seasons, which exhibited the highest risk in winter and the lowest risk in autumn. NO_2_ had the greatest effect on non-accidental mortality among all the pollutants, with RRs of 1.11 (95% CI: 1.01–1.21), 1.02 (95% CI: 0.85–1.23), 0.97 (95% CI: 0.75–1.25), and 1.19 (95% CI: 1.01–1.39) in spring, summer, autumn, and winter for every 10 μg/m^3^ increase in NO_2_. On the contrary, the cumulative effect of O_3_ on non-accidental mortality was highest in the spring and lowest in the summer; the difference was not significant in other seasons. Overall, air pollutants increased the mortality risk in the winter and spring as indicated by generally increased RR values.

### 3.5. Effects of temperature and air pollutants on the non-accidental mortality of subgroup populations

Table 4 summarizes the effects of temperature and air pollutants on the non-accidental mortality of different subgroups. The maximum mortality risk caused by the individual and combined effects of temperature and air pollutants over the 14-day lag period for each subgroup was calculated. In gender classification, both males and females were susceptible to temperature and air pollutants. Among them, males were more susceptible to the effects of temperature and temperature-air pollutant interactions; the maximum RR was highest at lag 0 day at respective values of 1.22 and 1.30. Females were more susceptible to the effects of NO_2_ and SO_2_; the maximum RR was highest over lag 0–2 days at 1.21 and 1.30, respectively. In terms of work environment, outdoor workers were more affected by temperature and air pollutants than indoor workers due to greater exposure. Both outdoor workers and indoor workers were more susceptible to temperature-air pollutant interactions, with the strongest effects on lag day 0 (RR = 1.29, 1.19). PM_2.5_ had the longest lag time for outdoor workers and was strongest on lag day 8 with an RR of 1.12 and lasting for about 12 days. SO_2_ had the longest lag time for indoor workers and was the strongest on lag day 5 (RR = 1.17) and lasting for about 9 days. The effect analysis on non-accidental mortality by age group showed that the older adults (≥75) were most affected by temperature and air pollution, and the RR of the temperature-air pollutant interactions was highest (1.31). People aged 60–74 years were more likely to be affected by temperature and temperature-air pollutant interactions, which reached a maximum on day 0 of lag (RR = 1.23). People aged 0–59 years were more susceptible to SO_2_ (RR = 1.26). Overall, temperature and air pollutants had a significant impact on non-accidental mortality in Rencheng, most highly affecting outdoor workers and the older adults.

**Table 4:** The effects of temperature and air pollutants over the lag 14-day maximum RR of different groups.

Effect subgroups	T_ave_ + PM_2.5_ + NO_2_ +SO_2_ + O_3_ (°C)		T_ave_ (°C)		PM_2.5_ (μg/m^3^)		NO_2_ (μg/m^3^)		SO_2_ (μg/m^3^)		O_3_ (μg/m^3^)	
	Maximum RR		Maximum RR		Maximum RR		Maximum RR		Maximum RR		Maximum RR	
	T_ave_	RR	T_ave_	RR	PM_2.5_	RR	NO_2_	RR	SO_2_	RR	O_3_	RR
Male	32	**1.30 (lag = 0)**	32	1.22 (lag = 0)	90	1.09 (lag = 0)	90	1.10 (lag = 0)	24	1.10 (lag = 0)	190	1.06 (lag = 0)
Female	32	1.17 (lag = 0)	32	1.12 (lag = 0)	160	1.09 (lag = 0)	90	1.21 (lag = 0)	74	**1.30 (lag = 2)**	120	1.05 (lag = 0)
Indoor worker	32	**1.19 (lag = 0)**	−11	1.10 (lag = 2)	85	1.08 (lag = 0)	90	1.12 (lag = 0)	74	1.17 (lag = 5)	170	1.03 (lag = 0)
Outdoor worker	32	**1.29 (lag = 0)**	32	1.22 (lag = 0)	240	1.12 (lag = 8)	90	1.16 (lag = 0)	74	1.26 (lag = 2)	135	1.08 (lag = 0)
0–59 years	32	1.06 (lag = 14)	−11	1.10 (lag = 2)	240	1.08 (lag = 5)	90	1.13 (lag = 0)	74	**1.26 (lag = 0)**	140	1.05 (lag = 11)
60–74 years	32	**1.23 (lag = 0)**	32	1.17 (lag = 0)	240	1.09 (lag = 3)	90	1.06 (lag = 2)	74	1.13 (lag = 3)	190	1.07 (lag = 14)
≥75 years	32	**1.31 (lag = 0)**	32	1.26 (lag = 0)	125	1.12 (lag = 0)	90	1.18 (lag = 0)	74	1.26 (lag = 2)	190	1.10 (lag = 0)

Bold values represent for the maximum values of the relative risk for different populations exposed to temperature and air pollutants.

## 4. Discussion

In this study, we examined the effects of temperatures and air pollutants on non-accidental mortality in the Rencheng District, China, 2019–2021. Our results indicated that both extreme cold/heat waves and air pollutants increase the risk of non-accidental deaths in the study area, with clear seasonal effects. The vulnerability of populations to temperatures and air pollutants varied depending on gender, working environment, and age.

### 4.1. Seasonal differences in non-accidental mortality

#### 4.1.1. Seasonal characteristics of temperatures on non-accidental deaths

The results showed that the pattern of temperature action differed in the winter and summer. In winter, the cold effect started late with a lag of about 1 day but lasted up to 12 days; in summer, the heat effect started on the same lag day but disappeared after about 3 days, similar to the results of existing studies (36). Chen et al. (37) developed a DLNM model to fit the relationship between temperature and non-accidental deaths in 272 major cities in China, showing that low-temperature effects lasted more than 14 days, while high-temperature effects lasted 2–3 days, consistent with the results of the present study. The results of this study suggested that relevant government departments should distinguish high and low temperatures when dealing with extreme temperatures. Low-temperature warning response measures should be relatively durable in the winter, and high-temperature warnings should be established early in the summer. The effect of temperature was larger in the summer than in the winter, which may be due to the fact that Rencheng is located in the northern region of China, where the centralized heating system is quite mature in the winter. Consequently, the local residents are not easily affected by extreme low temperatures. However, their heat tolerance might be relatively poor, making them more sensitive to extreme high temperatures. Residents living in northern areas may have a higher risk of mortality during hot weather than those living in southern areas (38, 39). The present study suggests that the Rencheng District should focus on response to extreme heat events for local designation of relevant public health policies and reducing disease burdens.

In addition, Figures 3A,B show that the RR value of 28.5°C is higher than the RR value of −16.2°C; the RR value of −7.2°C is close to the RR value of 38°C. The reasons for this result are: (1) Figure 3A shows the influence of daily minimum temperature (T_min_) on non-accidental death. From 2019 to 2021, the highest temperature in the T_min_ series was 28.5°C, and the corresponding maximum temperature was 37.4°C, which easily cause serious harm to human health. Meanwhile, the lowest temperature in the T_min_ series was −16.2°C, and the highest temperature on the corresponding date was −1.1°C. In addition, Jining City is located in the northern region of China, and the heating system in winter is relatively mature, so that the local residents have stronger cold tolerance and are less affected by low temperature. Taking all the above into consideration, it seems reasonable in Figure 3A that the relative risk of daily minimum temperature of 28.5°C is greater than the relative risk of daily minimum temperature of −15°C. (2) Figure 3B shows the effect of daily maximum temperature (T_max_) on non-accidental mortality. From 2019 to 2021, the minimum temperature in the T_max_ sequence was −7.2°C, and the minimum temperature in the corresponding date was −15.7°C, which easily cause serious harm to human health. Meanwhile, the maximum temperature in the T_max_ sequence was 38°C, and the minimum temperature in the corresponding date was 26.3°C, which had a relatively low impact on human health. Therefore, Figure 3B shows that the RR value of −7.2° C is close to the RR value of 38°C.

#### 4.1.2. Seasonal characteristics of air pollutants on non-accidental deaths

In our study, PM_2.5_, NO_2_, and SO_2_ were positively correlated with the number of non-accidental deaths; the impact on non-accidental deaths was highest in the winter, similar to existing research results (40). Qian et al. (20) also reported that the effects of PM_10_, SO_2_, and NO_2_ on non-accidental deaths were most consistent and obvious in the winter. However, the results were inconsistent with studies that found a stronger association between air pollution and warm-season mortality (41). Some reasons for the different results are as follows. The cold period of Jining City is from November to March of the following year. Due to an increase in coal combustion, the concentration of pollutants in the winter is highest and the PM_2.5_ concentration is about three times that of the summer. In addition, Jining City is located in mid-latitudes and is positioned in a temperate continental monsoon climate. In winter, it is cold and dry, with frequent adverse weather such as stagnant air, temperature inversion, and fog, hindering pollutant dissipation. On the contrary, Figure 2 shows that summer is a peak season for O_3_ concentration each year, followed by spring; however, the cumulative risk of O_3_ on non-accidental deaths was highest in spring, and the impact of other seasons was not obvious. This result was consistent with those of Chen et al. (42) on the impact of O_3_ on non-accidental deaths in Kunming from 2017 to 2019. However, contrary to the results of Chen et al. (43) on the impact of O_3_ on non-accidental deaths in Lishui City, summer had the greatest impact on non-accidental mortality. Studies have shown a negative correlation between PM_2.5_ and O_3_ (44); PM_2.5_ is the primary air pollutant in Rencheng District, and concentrations exceeded the national secondary standard level. In comparison, O_3_ concentration had no obvious effect on non-accidental deaths in the Rencheng District. Second, July–August in Jining is the rainy season. Rainfall plays a role in flushing and purifying air. However, there is less rainfall in spring, which shows a weak purification effect on air pollutants and may even experience aggravated air pollution. Relevant government departments should pay attention to the seasonal effects of air pollution. In order to reduce the concentrations of PM_2.5_, SO_2_, and NO_2_, the emission of automobile exhaust and coal burning should be controlled in winter and the emission of O_3_ should be reduced in spring.

### 4.2. Subgroup population analysis

In population stratification analysis, people older than 75 years and outdoor workers were more sensitive to extreme temperatures and air pollutants. Similar results have been found in previous studies (45). A study conducted in 16 Chinese cities between 1996 and 2008 also reported that older people were more vulnerable to extreme temperatures and pollutants (36). Consistent with our study, outdoor workers exposed to extreme temperatures and pollutants had a greater risk of all-cause mortality (46). The reason may be that certain physiological functions decline with age, particularly the immune systems, which can increase susceptibility to extreme temperatures and air pollution. In addition, outdoor workers are usually exposed to higher concentrations of air pollutants, resulting in greater health effects. The results showed that people aged 0–59 were extremely sensitive to SO_2_, which may also be caused by a small sample size needing further analysis and discussion. Regarding gender, women are more sensitive to air pollutant exposure and men are more sensitive to the effects of temperature and temperature-air pollutant interactions. Several studies have reported that women are more sensitive to air pollutants and have fewer red blood cells to metabolize the toxicity of pollutants than men (47). In addition, females have higher gas sensitivity, shorter respiratory tracts, and are more susceptible to air pollutants compared to males (48).

However, there were some limitations in this study. Only the data from the Rencheng District between 2019 and 2021 were collected and analyzed in this study, and the sample size was relatively small, especially that of people aged 0–59 years. It is necessary to increase data collection period in the future. In addition, this is an ecological study and there is no individual information such as smoking, drinking, and previous medical history, which may have some influence on the results.

## 5. Conclusion

In conclusion, extreme temperature and air pollutants were shown to increase the risk of non-accidental deaths in the Rencheng District. The incidence of non-accidental death was higher and the lag time was shorter in the warm season. Those older than 75 years and outdoor workers were more sensitive to extreme temperatures and air pollutants than younger people and indoor workers. Results in this study provide useful information for local governments and other countries with similar climate characteristics to develop relevant preventive interventions and improve worldwide public health strategies.

## Data availability statement

The original contributions presented in the study are included in the article/supplementary material, further inquiries can be directed to the corresponding authors.

## Author contributions

DY and SC: conceptualization, methodology, and writing—original draft preparation. DY: software, formal analysis, resources, and data curation. DY and SK: investigation. S-BL and SK: writing—review and editing. SX: visualization. SK: supervision. SC: project administration and validation. SC and SK: funding acquisition. All authors contributed to the article and approved the submitted version.

## Funding

This study was supported by the Humanities and Social Science Research Program funded by the Ministry of Education of China (21YJCZH010). SK’s research was supported by the Basic Science Research Program through the National Research Foundation of Korea (NRF) funded by the Ministry of Education (NRF-2021R1A2C1005271).

## Conflict of interest

The authors declare that the research was conducted in the absence of any commercial or financial relationships that could be construed as a potential conflict of interest.

## Publisher’s note

All claims expressed in this article are solely those of the authors and do not necessarily represent those of their affiliated organizations, or those of the publisher, the editors and the reviewers. Any product that may be evaluated in this article, or claim that may be made by its manufacturer, is not guaranteed or endorsed by the publisher.

## Footnotes

^1^http://www.cnemc.cn/sssj/

^2^https://data.cma.cn/
